# The Lnc RNA SPRY4-IT1 Modulates Trophoblast Cell Invasion and Migration by Affecting the Epithelial-Mesenchymal Transition

**DOI:** 10.1038/srep37183

**Published:** 2016-11-17

**Authors:** Qing Zuo, Shiyun Huang, Yanfen Zou, Yetao Xu, Ziyan Jiang, Shan Zou, Haoqing Xu, Lizhou Sun

**Affiliations:** 1 Department of Obstetrics and Gynecology, The First Affiliated Hospital of Nanjing Medical University, Nanjing, JiangSu Province, 210029, China; 2 Departments of Obstetrics and Gynecology, Yuhuangding Hospital of Yantai, Yantai, Shandong Province, 264000, China; 3 The Family Planning Science and Technology Research Institute of Jiangsu province, Nanjing, JiangSu Province, 210029, China

## Abstract

Preeclampsia is a common, pregnancy-specific disease and a major contributor to maternal and foetal morbidity and mortality. Some placental abnormalities, including deficient implantation, abnormal trophoblast cell function, and improper placental vascular development, are believed to lead to preeclampsia. The long noncoding RNA SPRY4-IT1 is more highly expressed in preeclamptic human placentas than in normal placentas. We assessed the role of epithelial-mesenchymal transition (EMT)-associated invasion and migration in HTR-8/SVneo trophoblast cells. Overexpression of SPRY4-IT1 suppressed trophoblast cell migration and invasion, whereas reduced expression of SPRY4-IT1 prevented the EMT process. Mechanistically, an RNA immunoprecipitation experiment showed that SPRY4-IT1 bound directly to HuR and mediated the β-catenin expression associated with EMT in HTR-8/SVneo cells. Moreover, the expression levels of genes in the WNT family, such as WNT3 and WNT5B, were changed after transfection of HTR-8/SVneo with SPRY4-IT1. Together, our results highlight the roles of SPRY4-IT1 in causing trophoblast cell dysfunction by acting through the Wnt/β-catenin pathway, and consequently in impairing spiral artery remodelling. These results suggest a new potential therapeutic target for intervention against preeclampsia.

Preeclampsia (PE) is a pregnancy-specific disease characterized by the occurrence of hypertension and proteinuria after 20 weeks of gestation in previously normotensive women^1^. It afflicts 3–5% of pregnancies and remains a leading cause of maternal mortality and morbidity worldwide, especially in developing countries^2^,^3^. The placenta is the key organ in the pathogenesis of PE, and its development is critical for embryonic development and successful pregnancy outcomes^3^. Impaired spiral artery remodelling, oxygen dysregulation, inappropriate maternal vascular damage and anomalous maternal-foetal inflammation-immune interactions^4^,^5^ are involved in the pathogenesis of this disease.

Among these characteristics, poor spiral artery remodelling has been considered to be a crucial early defect that causes PE and foetal growth restriction^6^. In pregnancy, to establish appropriate nutrient and oxygen supplies for the foetus, the extra-villous trophoblasts (EVTs) migrate through the endometrium, invade the uterine decidua, meeting only little resistance, and remodel the spiral arteries; this process is critical for the success of pregnanc^7^,^8^,^9^. EVTs are the most important functional cells in the placenta, and the abnormal migration and invasion of EVTs are pivotal contributors to the failure of placentation^10^,^11^. Many regulators affect EVT migration and invasion, including COX-2, PEG-2, MMP-2, and MMP-9, and cell signalling molecules such as Wnt/β-catenin and TGF-β^12^,^13^,^14^. The acquisition of migratory and invasive phenotypes by trophoblasts is an important aspect of the epithelial-to-mesenchymal transition (EMT)^11^.

The EMT refers to the conversion of epithelial cells to mesenchymal cells, which is crucial in the differentiation of multiple tissues and organs. The hallmarks of the EMT are a loss of E-cadherin and β-catenin expression and an increase in non-epithelial cadherins, such as N-cadherin and vimentin^15^. The cadherin/catenin complex actively participates in the EMT, which is physiologically and pathologically important^16^. For example, the EMT is involved in early stages of embryonic development, carcinogenesis, and wound healing. During wound healing, keratinocytes at the border of the wound recapitulate part of the EMT process^17^,^18^. The EMT has been identified as playing a key role in the metastasis of various carcinomas through regulating the migration and invasion potential of cancer cells^18^,^19^. Strikingly, EVTs display a phenotype very similar to that of cancer cells, regarding their capacity for proliferation, migration, and invasion^20^,^21^.

Recently, evidence has revealed that long noncoding RNAs (lncRNAs), such as the lncRNA HOTAIR^20^ (ID: 100124700) and MALAT1^22^ (ID: 378938), regulate the EMT process. In addition, Lan Xiao *et al.* have shown that SPRY4-I1 is required for HuR binding to RNA; it also directly interacts with tight junction mRNAs and consequently regulates intestinal epithelial barrier function^23^ Moreover, in human placental tissues, SPRY4-IT1 exhibits differential expression in severe PE placenta, and it contributes to the biological activities of trophoblasts^24^. On the basis of these findings and according to the crucial influence of trophoblast migration and invasion, we further investigated the potential molecular mechanism by which SPRY4-IT1 regulates spiral artery remodelling in PE. In the present study, we found that SPRY4-IT1 inhibits trophoblast cell migration and invasion partly via regulating the EMT process and may affect Wnt/β-catenin signalling.

## Results

### Upregulated expression of the long noncoding RNA SPRY4-IT1 in preeclamptic placentas

Clinical data were obtained from all patients who participated in our study. We classified the placental tissue into two groups: PE (n = 50) and normal pregnancy (n = 50). PE was diagnosed on the basis of a systolic blood pressure of ≥140 mm Hg after 20 weeks of gestation. Additionally, the two groups did not have any other complications, including a maternal history of hypertension and/or renal disease, severe intrauterine growth retardation, diabetes, alcoholism, smoking, chemical dependency, and foetal congenital abnormalities (Table 1).

**Figure 1:**
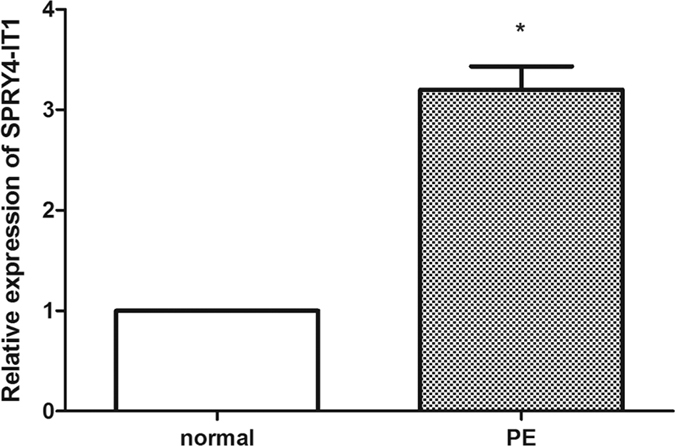
Inc RNA SPRY4-IT1 expression is increased in PE placentas. The relative expression of lncRNA SPRY4-IT1 was assessed by qRT-PCR using SYBR green and normalized to GAPDH. The levels of SPRY4-IT1 were lower in preeclamptic placentas (n = 50) than that in normal placentas (n = 50).

A qRT-PCR analysis was conducted to investigate the level of lncRNA SPRY4-IT1 expression in 50 placental tissues from women with PE and those from healthy women. Notably, the expression of SPRY4-IT1 was higher in pregnant women with PE than that in controls (Fig. 1). There were no significant differences in gestation age between the normal-pregnancy and PE groups (*P* > 0.05).

### Overexpression and knockdown of SPRY4-IT1 in human trophoblast cells (HTR-8/SVneo)

**Figure 2:**
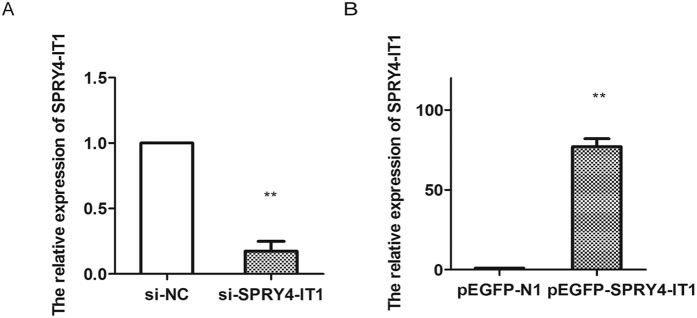
The transfection efficiency of si-SPRY4-IT1 and pEGFP-SPRY4-IT1. The transfection efficiency was assessed by qPCR. The relative expression of SPRY4-IT1 decreased 90% after transfection of si-SPRY4-IT1 (**A**) and the relative expression of SPRY4-IT1 was increased 82-fold after transfection of pEGFP-SPRY4-IT1 (**B**).

The significantly increased expression of SPRY4-IT1 in PE samples prompted us to explore the possible biological role of SPRY4-IT1. To assess the potential functions of SPRY4-IT1 *in vitro*, we used the trophoblast cell line HTR-8/SVneo. To manipulate the SPRY4-IT1 levels in trophoblast cells, we transfected HTR-8/SVneo cells with plasmids expressing SPRY4-IT1 and si-SPRY4-IT1 and then assessed SPRY4-IT1 expression 48 h later. Transfection of si-SPRY4-IT1 resulted in a 90% decrease in SPRY4-IT1 levels compared with that in the negative control. Furthermore, transfection of pEGFP-SPRY4-IT1 resulted in an 82-fold increase in SPRY4-IT1 levels compared with that of pEGFP-N1 (Fig. 2).

### SPRY4-IT1 inhibits the migration and invasion of HTR-8/SVneo cells

**Figure 3:**
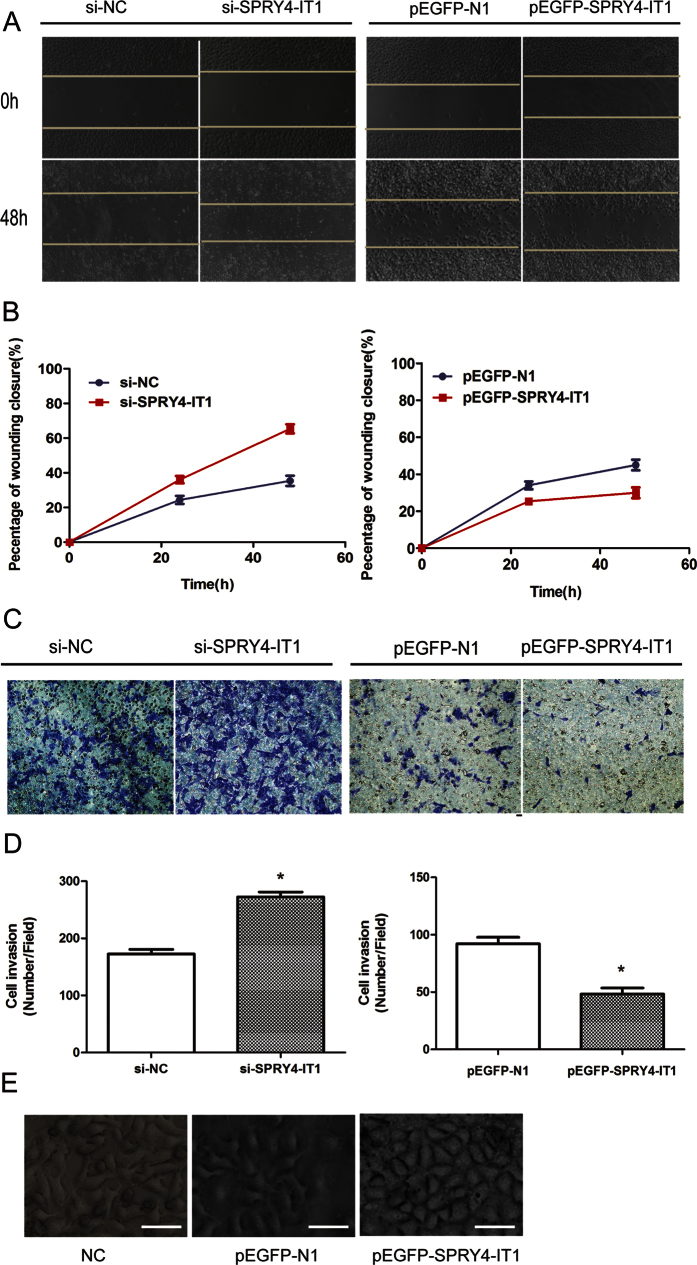
SPRY4-IT1 inhibits the migration and invasion of HTR-8/SVneo cells. (**A**) A wound-healing assay showed that scratch wounds closed more slowly in cells transfected with pEGFP-SPRY4-IT1 compared with controls, whereas they closed more quickly in cells transfected with si-SPRY4-IT1. (**B**) The percentage of the wound closure from the wound-healing assay. (**C**) The invasion capacity of the cells after knockdown and overexpression of SPRY4-IT1 as determined by Transwell assays (Values are mean ± SD; *P < 0.05; **P < 0.01). (**D**) The columns indicate the average number of cells from five fields. (**E**) SPRY4-IT1-silenced cells exhibited a more fibroblast-like shape. Cell morphology of silenced-SPRY4-IT1 HTR-8/SVeno cells and control cells Magnification, 400×; scale bars, 40 μm.

A wound-healing assay showed that scratch wounds closed more quickly in cells transfected with si-SPRY4-IT1 (Fig. 3A) than in control cells, whereas they closed more slowly in cells transfected with pEGFP-SPRY4-IT1 (Fig. 3B). We then used Matrigel assays to evaluate the invasive ability of trophoblast cells. The invasive ability was reduced after up-regulation of SPRY4-IT1 expression in HTR-8/SVneo cells, whereas increased SPRY4-IT1 expression levels impeded the migration of HTR-8/SVneo cells by approximately 1.6-fold compared with that of controls, as shown in the Transwell assays (Fig. 3C,D). Interestingly, SPRY4-IT1-overexpressing HTR-8/SVneo cells lost their fibroblast-like appearance (elongated and centrally located nuclei) and acquired a more epithelial morphology. In contrast, SPRY4-IT1-silenced HTR-8/SVneo cells exhibited a more fibroblast-like shape (Fig. 3E).

### Effects of SPRY4-IT1 expression on the EMT in HTR-8/SVneo cells

**Figure 4:**
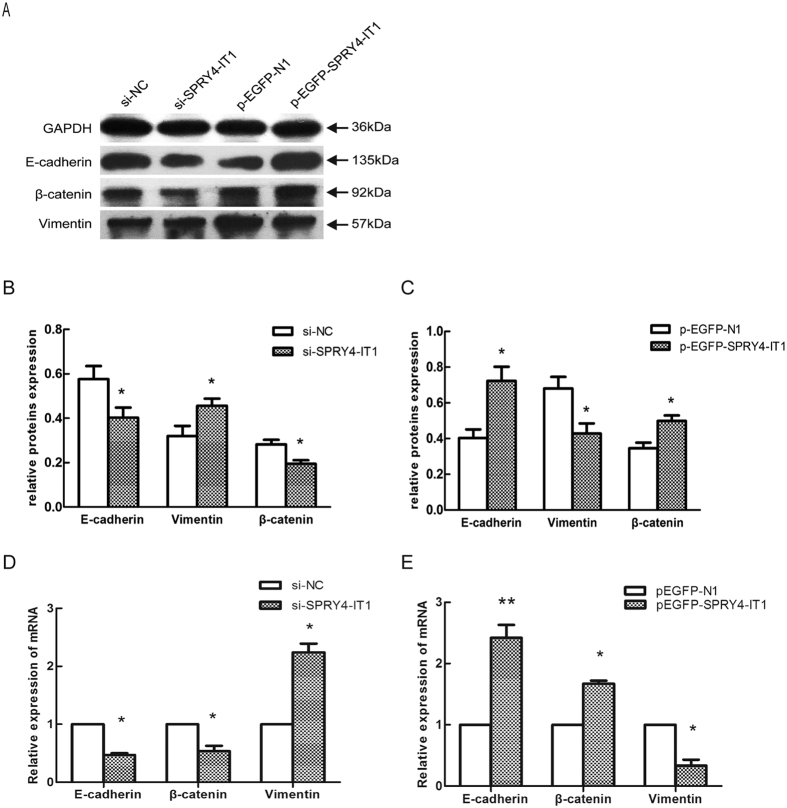
Changes in EMT markers after overexpression or knockdown of SPRY4-IT1 in HTR-8/SVneo cells. (**A**) Western blot assay of E-cadherin, β-catenin, and vimentin expression after SPRY4-IT1 was overexpressed or knocked down in HTR-8/SVneo cells. GAPDH protein was used as an internal control. (**B**,**C**) The columns indicate the relative expression of E-cadherin, β-catenin, and vimentin in HTR-8/SVneo cells in the western blot assay. (Values are means ± SD *P < 0.05; **P < 0.01). (**D**) The expression of E-cadherin, β-catenin, and vimentin detected by qRT- PCR after SPRY4-IT1 was knocked down in cells transfected with si-NC or si-SPRY4-IT1 or overexpressed in cells transfected with pEGFP-SPRY4-IT1 (**E**).

**Figure 5:**
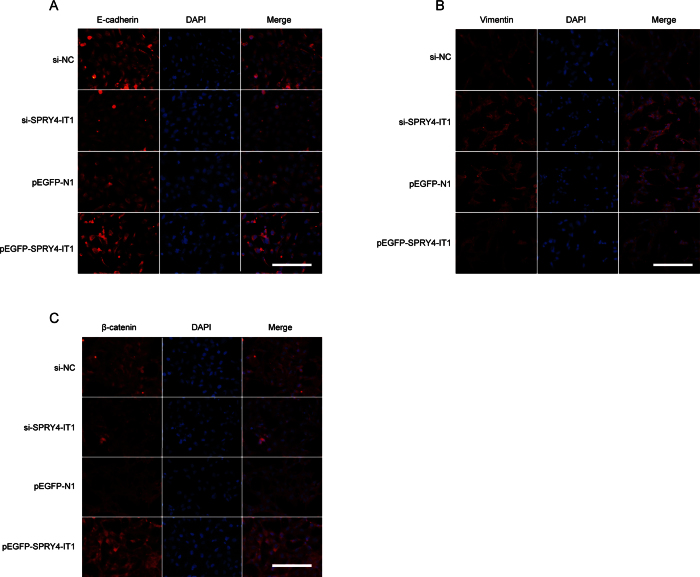
Changes in EMT-induced markers in HTR-8/SVneo cells, determined by immunofluorescence analysis. The immunofluorescence analysis of EMT-induced markers after SPRY4-IT1 was overexpressed or knocked down in HTR-8/SVneo cells. E-cadherin (**A**) and β-catenin (**C**) expression were down-regulated after transfection with si-SPRY4-IT1 compared with si-NC and up-regulated after transfection with pEGFP-SPRY4-IT1 compared with the empty vector. Vimentin expression levels (**B**) showed the opposite effects. Magnification 200×; scale bars, 20 μm.

Previously, we have found that the EMT is involved in trophoblast cell invasion and migration. In the present study, we determined whether SPRY4-IT1 regulates the EMT in trophoblast cells. We used western blotting to detect the expression of the EMT-induced markers E-cadherin, β-catenin, and vimentin in HTR-8/SVneo cells in which SPRY4-IT1 was overexpressed or knocked down. SPTY4-IT1 induced E-cadherin and β-catenin expression and decreased vimentin expression, whereas decreased SPRY4-IT1 expression inhibited E-cadherin and β-catenin expression and promoted vimentin expression (Fig. 4A–C). Moreover, the qRT-PCR (Fig. 4D,E) and immunofluorescence (Fig. 5) analyses revealed the same results in HTR-8/SVneo cells.

### Expression of EMT-induced proteins in preeclamptic placentas

**Figure 6:**
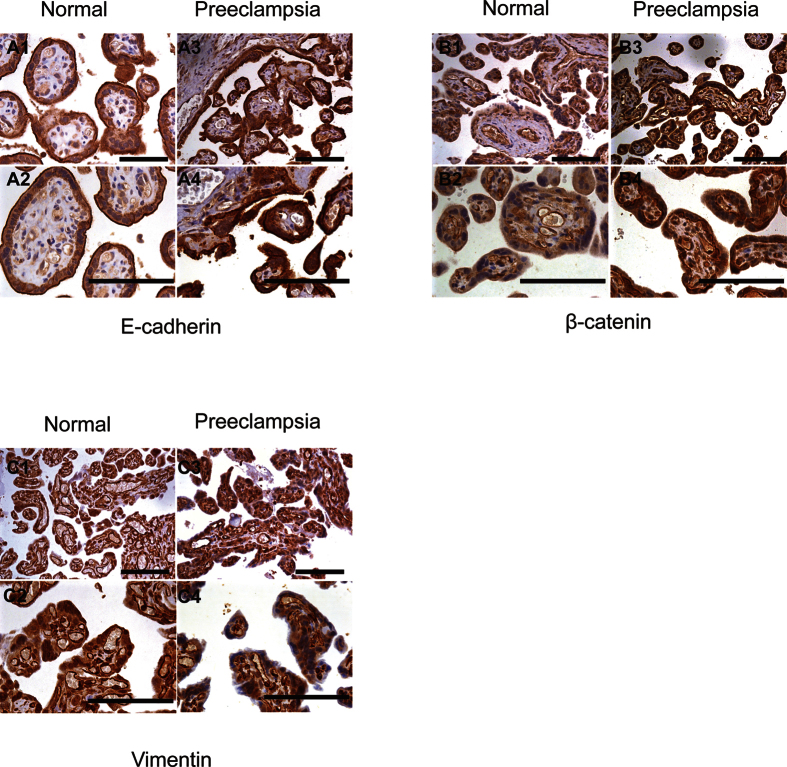
Immunohistochemistry of EMT-induced proteins in normal and preeclamptic placentas. Immunohistochemistry showing that E-cadherin (**A**) and β-catenin (**B**) expression in PE placenta are increased compared with that in normal placenta. The vimentin (**C**) expression showed the opposite effects. Magnification, 200×; scale bars, 20 μm (upper panels), 400×; scale bars, 40 μm (lower panels).

We performed immunostaining of the placental tissue from preeclamptic and normal pregnant woman to assess the presence and expression of EMT-induced markers E-cadherin, (Fig. 6A) β-catenin (Fig. 6B) and vimentin (Fig. 6C). Our results indicated that the staining intensity of E-cadherin and β-catenin in the placental tissue was stronger in the PE group than that in the control group. However, vimentin staining showed the opposite result, with lower staining in the PE group compared with the control group.

### SPRY4-IT1 mediates expression of the key downstream target β-catenin through binding to HuR

**Figure 7:**
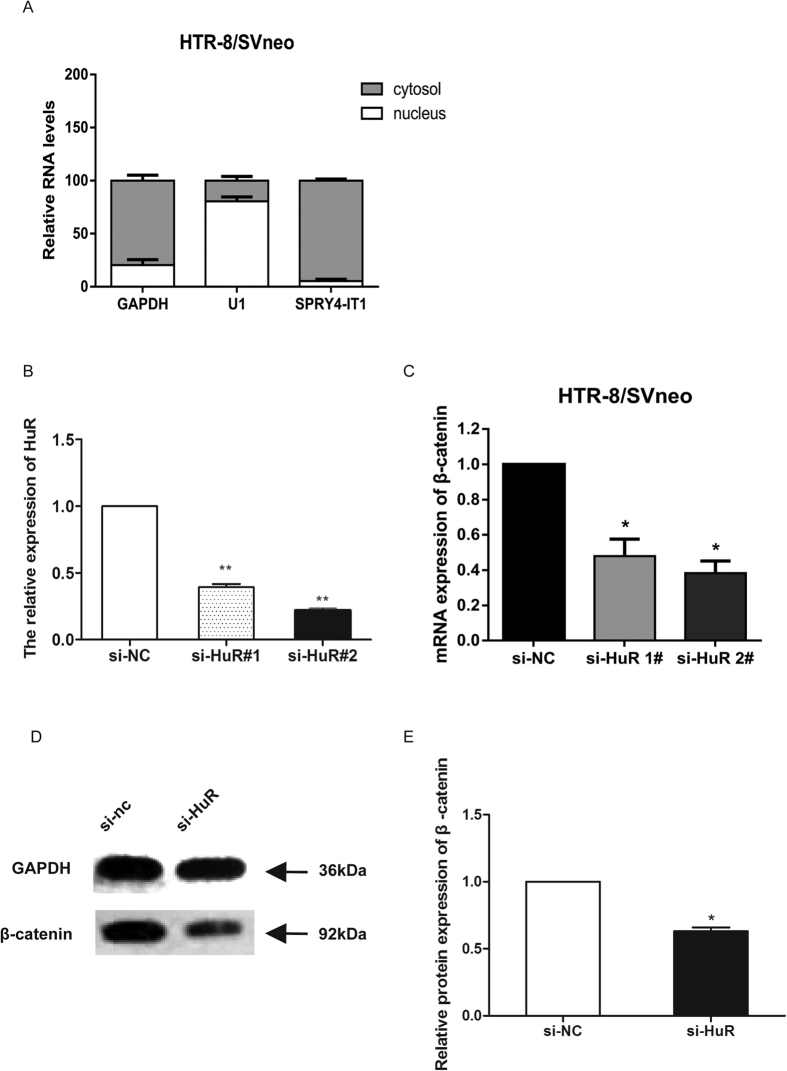
SPRY4-IT1 mediates expression of the key downstream target β-catenin through binding to HuR. (**A**) SPRY4-IT1 expression levels in different subcellular fractions in HTR-8/SVneo cells were detected by qRT-PCR. White range indicates the nuclear fraction, and the grey indicates the cytoplasmic fraction. (**B**) The relative expression of HuR decreased 65% and 81% after transfection of si-HuR. (**C**) The relative expression of β-catenin was significantly decreased in HTR-8/SVneo cells treated with HuR siRNA. (**D**) Western blot assay of β-catenin expression after HuR was knocked down in HTR-8/SVneo cells. GAPDH protein was used as an internal control. (**E**) The columns indicate the relative expression of β-catenin in HTR-8/SVneo cells transfected with si-HuR in the western blot assay. (Values are means ± SD *P < 0.05; **P < 0.01).

**Figure 8:**
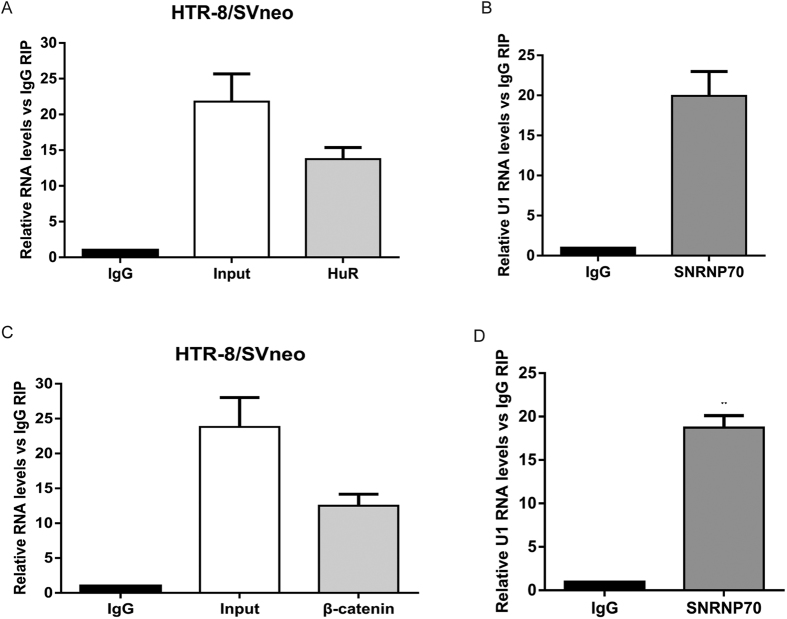
HuR directly binds to SPRY4-IT1 and β-catenin in HTR-8/SVneo cells. (**A**) RIP with rabbit monoclonal anti-HuR and preimmune IgG from HTR-8/SVneo cell extracts. RNA levels in immunoprecipitates were determined by qPCR. Expression levels of SPRY4-IT1 RNA are presented as fold-enrichment in HuR relative to IgG immunoprecipitates. (**B**) U1 binding with SNRNP70 is a positive control. (**C**) RIP with rabbit monoclonal anti-HuR and preimmune IgG from HTR-8/SVneo cell extracts. RNA levels in immunoprecipitates were determined by qPCR. Expression levels of β-catenin RNA are presented as fold-enrichment in HuR relative to IgG immunoprecipitates. (**D**) U1 binding with SNRNP70 is a positive control.

Our previous results indicated that SPRY4-IT1 overexpression increased the expression of β-catenin (Fig. 4). To investigate the underlying mechanism of SPRY4-IT1’s actions in trophoblast cells, we analysed the distribution of SPRY4-IT1 in HTR-8/SVneo cells. The results showed that approximately 91% of SPRY4-IT1 was distributed in the cytoplasm (Fig. 7A). Therefore, we speculated that SPRY4-IT1 might function at the post-transcriptional level in HTR-8/SVneo cells. According to previous reports, HuR is an important RNA-binding protein in the cytoplasm that modulates gene expression by altering the stability of mRNA. In addition, HuR has been reported to interact with β-catenin mRNA and to regulate its expression^25^,^26^,^27^,^28^. Thus, we hypothesized that SPRY4-IT1 might regulate the expression of β-catenin by interacting with HuR in HTR-8/SVneo cells. To verify that HuR regulated β-catenin expression, we treated HTR-8/SVneo cells with HuR siRNA (Fig. 7B). β-catenin expression was significantly lower in cells with HuR siRNA, as detected by qPCR (Fig. 7C). Similarly, western blot analysis showed the same results (Fig. 7D). Furthermore, we performed RIP assays which showed that SPRY4-IT1 directly bound HuR in HTR-8/SVneo cells (Fig. 8A), whereas it did not bind PRC2 and LSD1 (Figure S1); U1 binding to SNRNP70 was used as a positive control (Fig. 8B). Moreover, another RIP assay showed that HuR directly bound to β-catenin (Fig. 8C); U1 binding to SNRNP70 was used as a positive control (Fig. 8D). These data suggest that SPRY4-IT1 regulates β-catenin expression at the post-transcriptional level through binding to HuR.

### SPRY4-IT1 modulation of the *WNT*/β-catenin pathway

The effects of PE on Wnt-related gene expression in transfected HTR-8/SVneo cells were examined. β-catenin is the key transcriptional activator in canonical Wnt signalling in the nucleus, and HuR represses Wnt/β-catenin-mediated transcriptional activity by promoting the cytoplasmic localization of β-catenin^29^.

**Figure 9:**
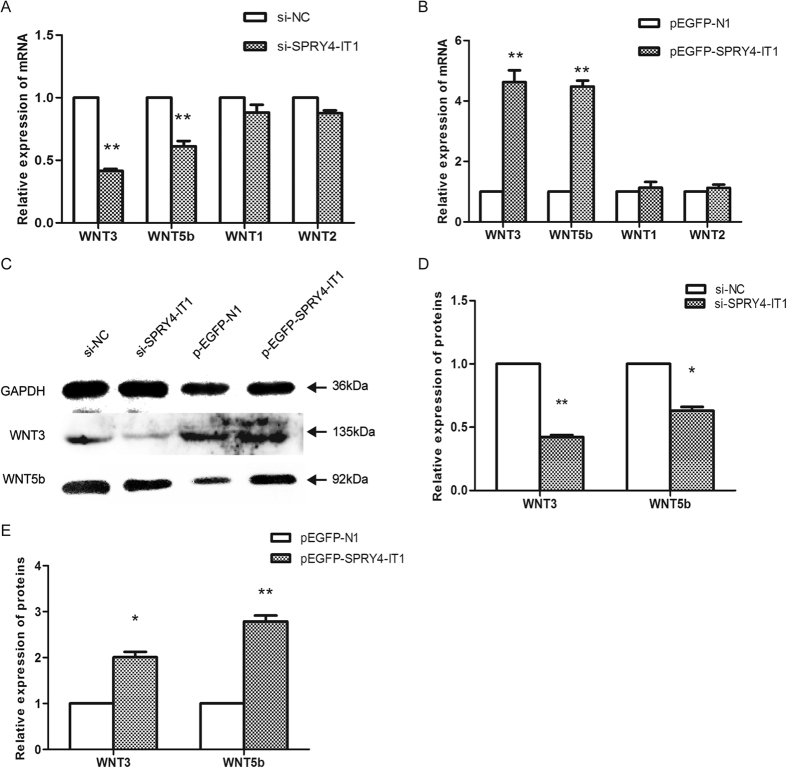
SPRY4-IT1 modulates the WNT/β-catenin pathway. The relative expression of Wnt3 and Wnt5b mRNA increased 4.6-fold and 4.5-fold, respectively after overexpression of SPRY4-IT1 (**B**), whereas it decreased 58% and 39% after SPRY4-IT1 knockdown (**A**), as determined by qRT-PCR. (**C**) Western blot assay of Wnt3 and Wnt5b expression after SPRY4-IT1 was overexpressed or knocked down in HTR-8/SVneo cells. GAPDH protein is an internal control. The columns indicate the relative expression of Wnt3 and Wnt5b in HTR-8/SVneo cells transfected with si-SPRY4-IT1 (**D**) and pEGFP-SPRY4-IT1 (**E**) in the western blot assay. (Values are means ± SD *P < 0.05; **P < 0.01).

We evaluated mRNA levels of members of the WNT family that have been reported to be associated with the EMT process, including *WNT1*, *WNT2*, *WNT3*, and *WNT5b*. Among these, the relative expression levels of *WNT3* and *WNT5b* mRNA were decreased 58% and 39% after SPRY4-IT1 knockdown, respectively (Fig. 9A), whereas they increased 4.6-fold and 4.5-fold, respectively after overexpression of SPRY4-IT1 (Fig. 9B), as determined by qRT-PCR (Fig. 4D,E). Additionally, western blot analysis showed the same changes in Wnt3 and Wnt5b expression as detected in the qRT-PCR assay (Fig. 9C).

## Discussion

Genomic studies have indicated that less than 2% of the total human genome can be transcribed as protein coding genes. The other transcripts, including not only the well-known microRNAs but also the lncRNAs, are represented in the primary transcriptome of the human genome. LncRNAs have emerged as critical regulators of gene expression and key players in many diseases^30^,^31^. For example, the lncRNA NRF regulates programmed necrosis and myocardial injury during ischaemia and reperfusion by targeting miR-873; MEG3 promotes hepatic insulin resistance via increasing FoxO1 expression; and Lnc-CC3 increases metastasis in cervical cancer by increasing Slug expression^32^,^33^. Collectively, lncRNAs have been investigated in different physiological and pathological processes for their ability to influence cell growth, survival, migration, and invasion^18^,^19^,^34^. Recently, several reports have described the expression profiles of lncRNAs in PE and the association between lncRNAs and the pathology of PE^35^. In previous studies, we have found aberrant expression of the lncRNA SPRY4-IT1^24^ and MEG3 in PE placentas and have demonstrated their modulatory effects on various biological functions of trophoblast cells^36^. In the present study, which is based on our previous work, we further verified the inhibitory role of SPRY4-IT1 in EMT-associated events, including invasion and migration of trophoblast cells^24^. When we overexpressed SPRY4-IT1 in HTR-8/SVneo cells, migration and invasion were inhibited. In contrast, knockdown of SPRY4-IT resulted in the opposite effects.

Increasing evidence indicates that lncRNAs regulate cell phenotypes through the modulation of gene expression by different mechanisms, for example, by recruiting transcriptional activators or repressors, remodelling chromatin, assembling ribonucleoprotein (RNP) complexes, functioning as scaffolds for protein assembly, and modulating gene expression by altering the stability and translation of mRNAs and jointly working with RBPs^37^,^38^,^39^,^40^. In a previous study, SPRY4-IT1 has been reported to bind the lipid phosphatase lipin 2 and to modulate apoptosis by altering lipin 2–mediated lipid metabolism^41^; SPRY4-IT1 has also been reported to interact with HuR, and SPRY4-IT1/HuR associations have been shown to be essential for the binding of HuR to the mRNAs encoding tight junction proteins. However, whether SPRY4-IT1 regulates expression of underlying target genes in trophoblast cells through any mechanism has never been reported.

SPRY4-IT1 was distributed primarily in the cytoplasm of HTR-8/SVneo cells. Therefore, we hypothesized that SPRY4-IT1 might regulate gene expression of its downstream targets at the post-transcriptional level. Interestingly, we found that SPRY4-IT1 directly bound HuR in HTR-8/SVneo cells. Many reports have indicated that HuR is an important RNA-binding protein in the cytoplasm that regulates multiple post-transcriptional processes including RNA stability and binds β-catenin mRNA and regulates its expression by altering its stability^42^,^43^,^44^. Along with this evidence, our investigations indicated that HuR bound β-catenin mRNA in HTR-8/SVneo cells. These findings indicated that lncRNA SPRY4-IT1 regulates β-catenin expression by binding to HuR in trophoblast cells.

The EMT process is generally associated with the generation and development of a number of human diseases^34^. Because of the importance of the EMT programmes in normal development in the generation of tissues and organs, as well as in their roles in cancer, stringent regulatory mechanisms are needed^45^. Although some investigators have suggested that placental trophoblast cells change from a coherently attached phenotype to a migratory phenotype that invades maternal decidual and spiral arteries in a process that resembles other developmental EMTs, few studies have addressed this possibility in great detail^46^,^47^. We found that SPRY4-IT1 is the key factor in the EMT process in PE. Our results suggest that SPRY4-IT1 might be involved in the dysfunction of trophoblast cells through its direct binding to HuR and consequent modulation of β-catenin expression, which is related to the EMT. β-catenin is the key transcriptional activator in the canonical Wnt signalling pathway in the nucleus, and HuR represses Wnt/β-catenin-mediated transcriptional activity by promoting the cytoplasmic localization of β-catenin^48^. Therefore, EMT appears to be regulated by the Wnt/β-catenin pathway^49^,^50^. These results, combined with the change in WNT3 and WNT5B expression with alterations in SPRY4-IT1 expression in HTR-8/SVneo cells, indicate that SPRY4-IT1 might regulate the EMT in trophoblast cells through Wnt/β-catenin signalling.

In conclusion, our study indicates that the expression of SPRY4-IT1 is increased in preeclamptic placentas and may alter the biological functions of trophoblasts *in vitro*. Together, our results demonstrate that SPRY4-IT1 might be involved in the dysfunction of trophoblast cells through a potential molecular mechanism that is related to impair spiral artery remodelling. These data suggest that SPRY4-IT1 is an important mediator of the EMT and might be a promising therapeutic option for suppressing PE progression. The present study provides additional evidence supporting the activation of Wnt signalling in PE, but alternative mechanisms remain to be further clarified. Although our study provides new insight into the mechanisms of PE, there are a number of important mechanisms through which SPRY4-IT1 may perform a regulatory role in the EMT in addition to the Wnt/β-catenin pathway.

## Methods and Materials

### Tissue collection

We obtained 50 paired placentas from nulliparous women who underwent caesarean section during 2015 in the Department of Obstetrics and Gynaecology of People’s Hospital of Jiangsu Province, China. Tissues were collected from the mother surface of the placenta as well as near the root of the umbilical cord. All collected tissues were washed with sterile phosphate-buffered saline, then immediately snap-frozen in liquid nitrogen, and stored at −80 °C until needed. Experiments were approved by the Ethics Board of the First Affiliated Hospital of Nanjing Medical University, and written informed consent was obtained from each patient. All clinical investigations were conducted according to the principles expressed in the Declaration of Helsinki.

### Cell culture and treatment

HTR-8/SVneo cells (kindly provided by Dr. Charles Graham, Queen’s University, Canada)^51^, which were derived from a short-lived primary EVT cell line, were maintained in RPMI 1640 medium supplemented with 10% heat-inactivated foetal bovine serum (FBS), 100 μg/ml streptomycin and 100 U/ml penicillin (Gibco, Grand Island, NY, USA). Cells were grown under standard culture conditions at 37 °C in a humidified 5% CO_2_ incubator. Transient cell transfections of siRNA and plasmid vectors were performed with Lipofectamine RNAiMax (Invitrogen, Carlsbad, CA) and FuGENE HD Transfection reagent (Roche, Mannheim, Germany) according to the manufacturer’s instructions after being seeded in 6-well plates and grown overnight. Cells were harvested 48 h after transfection to detect the overexpression or knockdown of SPRY4-IT1. Three different Stealth siRNAs against SPRY4-IT1 were obtained from Invitrogen. The target sequences for SPRY4-IT1 were si-SPRY4-IT1-1 (CCCAGAATGTTGACAGCTGCCTCTT), si-SPRY4-IT1-2 (TGGAGGGTTATGGGAGCCTGTGAAT), and si-SPRY4-IT1-3 (GCTTTCTGATTCCAAGGCCTATTAA), and si-SPRY4-IT1-3 had the highest inhibition efficiency. The SPRY4-IT1 sequence (708 bp) was synthesized and cloned into the pEGFP-N1 plasmid vector (Invitrogen, Shanghai, China). Ectopic expression of SPRY4-IT1 (constructed by Invitrogen) was achieved through pEGFP-SPRY4-IT1 transfection, with an empty pEGFP-N1 vector used as control. Two different Stealth siRNAs against HuR were provided by Invitrogen. The target sequences for HuR were si-HuR-1 (UUACCAGUUUCAAUGGUCATT UGACCAUUGAAACUGGUAATT) and si-HuR-2 (CACGCUGAACGGCUUGAGGTTCCUCAAGCCGUUCAGCGUGTT), and the latter had the higher inhibition efficiency.

### RNA extraction and real-time RT-PCR

Total RNA was isolated with TRIzol reagent (Invitrogen) from the placental tissue or cells. A Reverse Transcription Kit (TaKaRa Bio, Shiga, Japan) was used for the synthesis of cDNA, and 1 mg total RNA was added to the RT Reaction Mix. SYBR Premix Ex Taq (TaKaRa Bio) was used to determine SPRY4-IT1 expression levels according the manufacturer’s instructions, and results were normalized to glyceraldehyde-3-phosphate dehydrogenase (GAPDH). qRT-PCR assays were performed with an ABI 7500 (Invitrogen). The results were analysed and expressed relative to threshold cycle (CT) values and then converted to fold changes.

### Western blotting analysis

The HTR-8/SVneo cells were transfected with knockdown and overexpression plasmids and prepared for protein extraction. We used mammalian reagent RIPA buffer (Beyotime Biotechnology, Shanghai, China), protease inhibitor cocktail (Roche, Pleasanton, CA, USA), and phenylmethylsulfonyl fluoride (Roche) to lyse the samples. The Bradford assay was used to determine the protein concentration in each sample. Protein extracts (50 μg) were resolved on 10% SDS-polyacrylamide gel electrophoresis, transferred to polyvinylidene difluoride membranes (Sigma-Aldrich, St. Louis, MO, USA), and incubated with antibodies against WNT3 and WNT5b (all purchased from Proteintech), E-cadherin, β-catenin and vimentin (all purchased from Cell Signaling Technology, Danvers, MA, USA) at a 1:1000 dilution. Autoradiograms were quantified by densitometry using Quantity One software (Bio-Rad, Hercules, CA, USA), using GAPDH as a control. The secondary antibody was horseradish peroxidase-conjugated goat anti-mouse or goat anti-rabbit IgG at a 1:1000 dilution.

### Immunofluorescence

Immunofluorescence assays were performed according to a previously published method^52^. Cells were incubated with rabbit antibodies against E-cadherin, β-catenin, and vimentin (all from Cell Signaling Technology and diluted 1:100) overnight at 4 °C. The cells were then incubated with FITC-conjugated secondary antibody (1:200; Proteintech, Chicago, IL, USA) at room temperature for 1 h and DAPI for 5 min. Images were acquired on an Olympus BX51 microscope (Tokyo, Japan).

### *In vitro* invasion assays

For the *in vitro* Transwell invasion assays, 6 × 10^4^ transfected HTR-8/SVneo cells were suspended in RPMI 1640 with 1% FBS and added to the upper chamber of each insert (8-μm pore size; EMD Millipore, Billerica, MA, USA) with a coated membrane. The chamber was coated by addition of 50 μL Matrigel (BD Bioscience, Franklin Lakes, NJ, and USA) into the upper chamber. In the lower chamber, 700 μL RPMI 1640 medium with 10% FBS was added. After incubation for 48 h at 37 °C and 5% CO_2_, cells that invaded through the membrane filter were fixed, stained with crystal violet, counted, and imaged through an Olympus IX71 inverted microscope (Tokyo, Japan). We counted cell numbers in at least five grids per field and calculated the average values. All assays were independently repeated three times.

### Wound-healing assay

Cells were seeded in six-well plates, transfected, and grown until 80–90% confluence. Wounds were created by scraping the plates with a pipette tip, and the debris was removed with PBS. Images were obtained to assess the ability of the cells to migrate into the wound area at the indicated time points. Each experiment was carried out in triplicate at least three times.

### Isolation of cytoplasmic and nuclear RNA

A PARIS Kit (Life Technologies, Carlsbad, CA, USA) was used to separate and purify the cytoplasmic and nuclear RNA. All steps were performed according to the manufacturers’ instructions. All assays were independently repeated three times.

### RNA- binding protein immunoprecipitation (RIP) assay

The RNA immunoprecipitation (RIP) assays were carried out with a Magna RIP™ RNA-Binding Protein Immunoprecipitation Kit (Millipore, USA). All steps were performed according to the manufacturer’s instructions. The antibodies for the RIP assay of EZH2, LSD1, HUR, and SUZ12 were purchased from Millipore. All assays were independently repeated three times.

### Immunohistochemistry

Placentas were fixed in 4% paraformaldehyde for 24–36 h following a standard protocol, then dehydrated and embedded in paraffin. Tissue sections (5-μm thickness) were mounted on glass slides (Thermo Scientific, Waltham, MA, USA). Rabbit anti-E-cadherin, anti-vimentin, and anti-β-cadherin polyclonal antibodies (1:100; Cell Signaling Technology) were used as primary antibodies, and anti-rabbit IgG (1:200; Sigma-Aldrich) used as a secondary antibody. Sections were mounted onto slides using Gel Mount Aqueous Mounting Medium (Sigma-Aldrich) and examined with an Olympus BX51 microscope.

### Statistical analysis

All data are expressed as the mean ± SD (standard deviation). Student’s *t*-test (two-tailed) and one-way ANOVA were used to evaluate the data with SPSS statistical software (SPSS Inc., Chicago, IL, USA). The data were considered to be statistically significant when *P*-values were less than 0.05.

## Additional Information

**How to cite this article**: Zuo, Q. *et al.* The Lnc RNA SPRY4-IT1 Modulates Trophoblast Cell Invasion and Migration by Affecting the Epithelial-Mesenchymal Transition. *Sci. Rep.* **6**, 37183; doi: 10.1038/srep37183 (2016).

**Publisher’s note:** Springer Nature remains neutral with regard to jurisdictional claims in published maps and institutional affiliations.

## Supplementary Material

Supplementary Dataset 1

Supplementary Information

## Figures and Tables

**Table 1:** Clinical characteristics of normal and preeclamptic pregnancies.

Variable	PE (n = 50)	N (n = 50)	P^a^ value
			Control vs PE
Maternal age	29.6 ± 4.7	30.4 ± 3.5	>0.05
Proteinuria (g/day)	>0.3	<0.3	<0.01
Gestational age (week)	35.8 ± 3.3	38.8 ± 1.3	>0.05
Systolic blood pressure, mm Hg	169 ± 16.8	104 ± 6.3	<0.01
Diastolic blood pressure, mm Hg	121 ± 12.2	69 ± 6.9	<0.01
Body weight of infant (g)	2493 ± 652	3501 ± 392	<0.05
CRP (C-reaction proteins)	7.1 ± 3.2	5.3 ± 2.3	>0.05
Maternal smoking (number)	3	1	>0.05

All results are presented as the mean ± SD. SD, standard deviation.

^a^Obtained by 1-way analysis of variance using SPSS 13.0 software (SPSS Inc, Chicago, IL).
