# Supplementary material for: The Lnc RNA SPRY4-IT1 Modulates Trophoblast Cell Invasion and Migration by Affecting the Epithelial-Mesenchymal Transition
Source: Sci Rep. 2016 Nov 17;6:37183. doi: 10.1038/srep37183 (PMC5112580; doi:10.1038/srep37183)
Supplement: Supplementary Information [file srep37183-s2.doc]

**The Lnc RNA SPRY4-IT1 Modulates Trophoblast Cell Invasion and Migration by Affecting the Epithelial-Mesenchymal Transition**

Qing Zuo^1^, Shiyun Huang^1^, Yanfen Zou^2^, Yetao Xu^1^, Ziyan Jiang^1^, Shan Zou^1^, Haoqing Xu^3^, Lizhou Sun^1^*

**Supplementary information**

Figure -S1 **PRC2 and LSD1 could not directly binds with SPRY4-IT1**


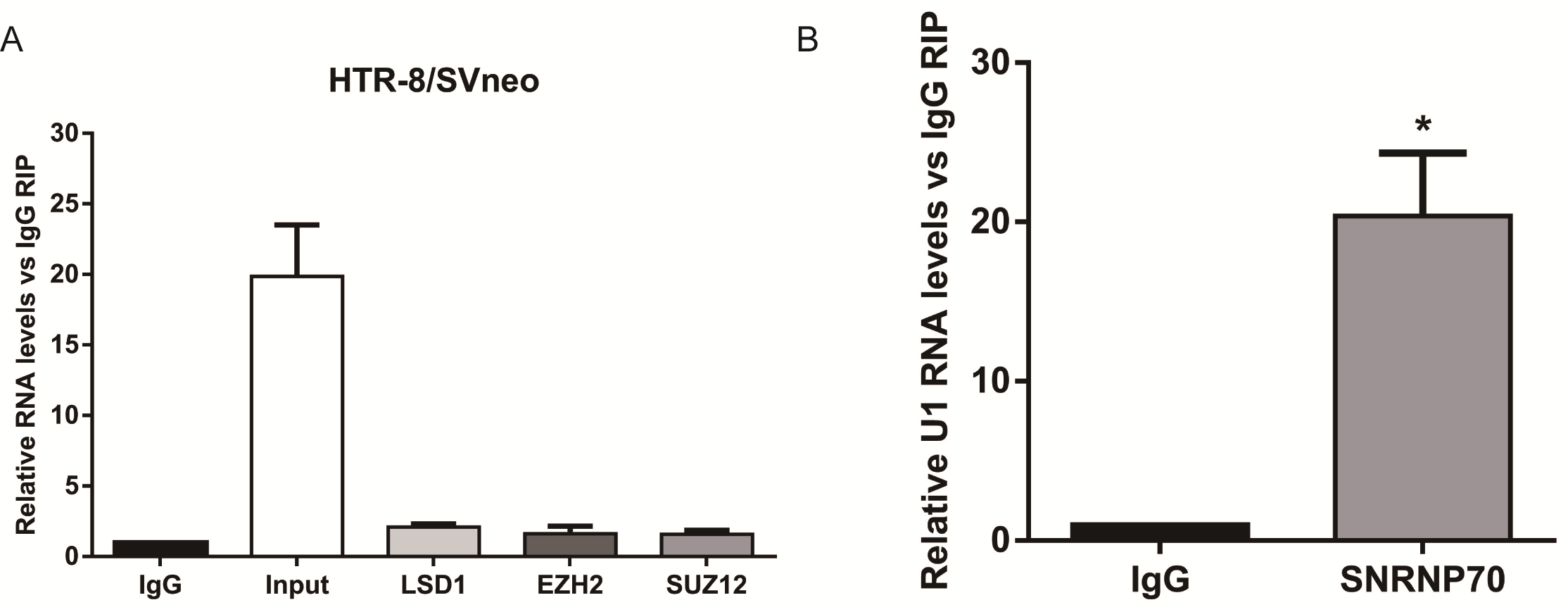


**PRC2 and LSD1** **could not directly binds with SPRY4-IT1**

RIP with rabbit monoclonal anti- PRC2 and LSD1 (A) and preimmune IgG from HTR-8/SVneo cells extracts. RNA levels in immunoprecipitates were determined by qPCR. Expression levels of SPRY4-IT1 RNA were presented as fold enrichment in HuR relative to IgG immunoprecipitates. SNRNP70 was used as positive control (B).
